# Vitamin A decreases the anabolic bone response to mechanical loading by suppressing bone formation

**DOI:** 10.1096/fj.201802040R

**Published:** 2019-01-22

**Authors:** Vikte Lionikaite, Petra Henning, Christina Drevinge, Furqan A. Shah, Anders Palmquist, Pernilla Wikström, Sara H. Windahl, Ulf H. Lerner

**Affiliations:** * Department of Internal Medicine and Clinical Nutrition, Centre for Bone and Arthritis Research, Institute for Medicine, Sahlgrenska Academy at University of Gothenburg, Gothenburg, Sweden;; † Department of Biomaterials, Institute of Clinical Sciences, Sahlgrenska Academy at University of Gothenburg, Gothenburg, Sweden; and; ‡ Department of Medical Bioscience, Pathology, Umeå University, Umeå, Sweden

**Keywords:** osteoblasts retinoids, physical activity, Raman spectroscopy

## Abstract

Increased vitamin A consumption is associated with decreased cortical bone mass and increased fracture risk in humans. Rodent studies have demonstrated that hypervitaminosis A increases cortical bone resorption, whereas the importance of the effects on bone formation is less well defined. We used an experimental model of increased bone formation by loading of the tibiae to investigate the effect of vitamin A on bone formation. Control [retinol activity equivalents (RAE) 4.5 µg/g chow] or vitamin A (RAE 60 µg/g chow) diets were given to female C57BL/6N mice for 4 wk, after which the tibiae were subjected to axial loading on alternate days for 2 wk, while the diets were continued. Vitamin A inhibited the loading-induced increase in trabecular and cortical bone volume. This was attributed to inhibition of loading-induced increase in osteoblast number and activity, and expression of osteoblastic genes *Sp7*, *Alpl*, and *Col1a1* in cortical bone. Vitamin A, loading, and combination thereof also resulted in site-specific effects on bone composition measured by Raman spectroscopy. In summary, a clinically relevant dose of vitamin A suppresses the loading-induced gain of bone mass by decreasing bone formation. These observations may have implications for regulation of bone mass caused by physical activity and the risk of osteoporosis in humans.—Lionikaite, V., Henning, P., Drevinge, C., Shah, F. A., Palmquist, A., Wikström, P., Windahl, S. H., Lerner, U. H. Vitamin A decreases the anabolic bone response to mechanical loading by suppressing bone formation.

Bone remodeling is a continuous process throughout life that is balanced by bone-forming osteoblasts and bone-resorbing osteoclasts (1, 2). With age, the balance of remodeling is often disrupted, and bone resorption exceeds formation, leading to decreased bone mass and, eventually, osteoporosis and fractures (3–5). Although preventative measures can be taken to delay the onset and magnitude of bone loss (*e.g.*, diet and exercise), bone loss can also be exacerbated by drugs such as glucocorticoids and vitamins such as vitamin A (retinol) if consumed in excess.

Vitamin A is found in foods such as meat, dairy products, and vegetables. A balanced diet is sufficient to maintain the nutritional needs; however, fortification of products as well as supplementation with vitamins leads to an increased risk of hypervitaminosis A and is becoming an increasing problem (6). Excess vitamin A consumption and elevated serum retinol levels have been associated with increased bone fragility and fracture risk in humans (7–10). This association indicates that increased vitamin A intake may be a risk factor for secondary osteoporosis.

The current recommended daily allowance for vitamin A consumption in adults is 900 and 700 µg retinol activity equivalents (RAE) per day in men and women, respectively (11). The upper tolerable limit of maximum vitamin A consumption that does not pose ill effects is 3000 µg/d (11). Supplements, whether single-ingredient or multimineral or multivitamin when combined with food or each other, often contain over 100% of the recommended daily allowance of 1 or more nutrients (12). Besides professional athletes (13), the elderly (aged 60 y and over) are the highest users of supplements (12). For this reason, supplementation of vitamin A or constituents high in vitamin A (*e.g.*, liver oil), in addition to an already balanced diet, may exacerbate bone loss.

In experimental rat studies, a 142-fold increase in vitamin A intake (RAE vitamin A 510 µg/g chow) has been illustrated to induce hypervitaminosis A and vitamin A toxicity determined by serum retinol status, reduced food intake, and reduction in weight gain (14–16). In rats receiving oral gavage of a 200–500-fold increase of vitamin A levels (RAE vitamin A 3000–7500 µg/d), spontaneous long-bone fractures have been reported (17). Short-term hypervitaminosis A in rodents decreases cortical bone because of an increased number of osteoclasts on the periosteal bone (14, 17–19) and a decreased number on the endocortical bone (14).

The effects of vitamin A on bone formation have been less well studied. In 2 studies, rats fed hypervitaminosis A diet containing 1700 IU (RAE vitamin A 510 µg/g chow) for 7 d have decreased osteoblast activity and number on the periosteal bone of the femur (15) and on the pericranial side of the calvaria (16). In another study, mice given daily injections of 125 µg/kg of the retinoid Ro 13-6295 for 4 d had a reduced number of osteoblasts with no effect on their activity (19).

Although the doses of vitamin A used in rodent studies are high, they are not necessarily reflective of human consumption in either quantity or duration. More recently, we have shown that a clinically relevant dose of vitamin A (RAE 60 µg/g chow), which is only 13 times higher than control diet, decreased periosteal bone formation after 1 wk and also increased endocortical bone formation after 1 and 4 wk of treatment in mice (20). Thus, *via* concomitant increase in bone resorption and decrease in bone formation, excess vitamin A can lead to decreased bone strength (14, 21) and increased risk of fractures (8, 9, 22–24).

Bone strength is dependent on size, architecture, and composition. Loading of the skeleton during physical activity leads to recruitment of bone-forming osteoblasts in order to adapt the bones to the applied strain, thereby increasing bone strength (25). Bone is composed of organic (mainly collagen type 1 fibers) and inorganic (hydroxyapatite, calcium, phosphate) compounds that reflect the quality of the bone. Axial mechanical loading of the tibia in rodents is the gold standard of studying bone response to load (26). It is also a good model of impact sports and can be used against a background of various dietary supplements. Often it is noted that the opportune time to enhance bone strength and reduce the risk of fractures later in life is during childhood and puberty; however, implementation of exercise in postmenopausal women has also shown increases in bone mineral density (BMD) at the lumbar spine and femoral neck (27–31).

We hypothesized that a clinically relevant dose of vitamin A may inhibit the bone-forming effects of mechanical loading in mice, in addition to activation of bone resorption. Therefore, we assessed the loading response in bone with and without prior and concurrent treatment with a clinically relevant dose of vitamin A.

## MATERIALS AND METHODS

### Animals

Animal experimental procedures were approved by the Ethics Committee at the University of Gothenburg and carried out in accordance to relevant guidelines. Female C57BL/6N mice (Taconic Biosciences, Ejby, Denmark) were received at 12–13 wk of age and acclimatized for 1 wk. At the start of experiment, mice were considered mature adults at 13–14 wk of age, which corresponds to a human age of 20–30 yr (32). Mice were housed in groups of 8–10 at 22°C with a 12-h light/dark cycle and fed diets (Envigo, Huntingdon, United Kingdom) containing either RAE vitamin A 4.5 µg/g chow (control, TD.00217; Envigo) or by adding vitamin A to the ground control chow and repelleted to make a final content of RAE 60 µg/g chow (vitamin A) *ad libitum*. The vitamin A was in the form of retinyl acetate. We have previously shown that this vitamin A dose decreased periosteal bone formation after 1 wk of treatment and increased endocortical bone formation after 1 and 4 wk of treatment (20). The vitamin A levels in control chow correspond to a daily intake of RAE 675–1125 µg/kg (using mice reference body weight of 20 g and food intake of 3–5 g/d chow). Based on body surface (33, 34), this dose would correspond to a human equivalent dose of RAE 56–91 µg/kg body weight. The vitamin A diet contains 13-fold more vitamin A, which is similar to what has been used in other studies of increased vitamin A (21, 35–37). This dose results in serum concentrations of 230 nM retinyl esters after 4 wk (20), which is just above the levels suggested to indicate excess vitamin A in humans (200 nM) (38). All mice were divided into body weight–matched groups at the start of the experiment. Power analysis suggested that, using 8 mice per group, we would have 80% power to detect a biologic significant effect with a 1.51 sd change in cortical bone thickness at a 2-sided α level of 0.05.

### Mechanical strain testing

**Figure 1:**
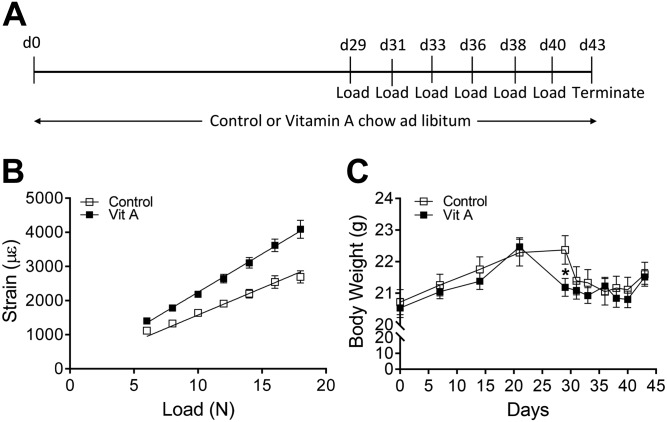
Experimental design, strain *vs.* load from *ex vivo* measurements, and body weight throughout loading. *A*) Timeline of the experiment. *B*) The effect of vitamin A on the strain and load relationship as determined by mechanical strain testing; *n* = 3–6 per group. *C*) Body weights throughout experiment; *P < 0.05 unpaired Student’s *t* test, *n* = 17 per group. Data in (*B*, *C*) are displayed as means ± sem.

Thirteen-week-old female mice were fed control or vitamin A diet for 4 wk prior to strain gauging (*n* = 3–6/group). The mechanical strain applied throughout the experiment was established *ex vivo* in postmortem intact mice with the tibia still attached to the body as previously described (39, 40). Briefly, a single-element strain gauge (EA-06-015DJ-120; Vishay Precision Group, Wendell, NC, USA) was longitudinally aligned to the diaphysis of the tibia at 37% of its length from the proximal end and was adhered with cyanoacrylate adhesive. A range of peak compressive loads was applied with a trapezoidal waveform using an ElectroForce 3100 Test Instrument (Bose, Eden Prairie, MN, USA) to measure the strain. Linear regression analysis allowed for calculation of peak load (**Fig. 1*B***). From these *ex vivo* data, specific peak loads of 14.4 and 10.1 N were used for control and vitamin A mice, respectively, during the *in vivo* loading experiment. These loads corresponded to 2250 µε at the gauge site, which is equivalent to the strain applied to the human tibia while jumping or zig-zag hopping on 1 leg (41, 42).

### *In vivo* tibia loading

Female 13-wk-old mice (*n* = 17/group) were fed control or vitamin A diets 4 wk prior to loading and throughout the duration of the loading procedure (Fig. 1*A*). While under inhalation anesthesia with isoflurane (Baxter, Deerfield, IL, USA), the right tibia of each mouse was placed in the holding cups of the ElectroForce 3100 Test Instrument. Axial load was applied through the knee joint for 40 cycles/d (trapezoid waveform) with 10 s rest between cycles on 3 alternative d/wk for 2 wk (40, 43). The left tibia was not manipulated and was used as a nonloaded, contralateral control. The mice were allowed free cage activity between the loading sessions. At termination of the experiment, the mice were euthanized by exsanguination under anesthesia followed by cervical dislocation, and their tibias were dissected and soft tissue removed. Bones were fixed in formalin for 48 h and thereafter stored in 70% ethanol or flushed of bone marrow and stored at −80°C in RNAlater (Qiagen, Hilden, Germany).

### Microcomputed tomography

Microcomputed tomography (µCT) analysis was performed on the tibiae using the Skyscan 1072 scanner (Bruker, Aartselaar, Belgium) and imaged with an X-ray tube voltage of 50 kV and current of 201 µA with a 0.5 mm aluminum filter. Scanning angular rotation was 180° with an angular increment of 0.70°. The voxel size was 4.48 µm isotropically. NRecon software (v.1.6.9; Bruker) was used for image reconstruction. CT Analyzer software (Bruker) was used for trabecular and cortical bone analysis. For trabecular bone parameters, trabecular bone distal to the proximal growth plate was selected for analysis within a conforming volume of interest (cortical bone excluded), commencing at a distance of 650 μm from the growth plate and extending a further longitudinal distance of 134 µm in the distal direction. For cortical bone, the diaphyseal region was analyzed 2.36 mm distal from the growth plate and extended a further longitudinal distance of 134 µm in the distal direction, corresponding to ∼37% of the length of the tibia from the proximal end. The equipment was calibrated with ceramic standard samples (calibration phantoms, calcium hydroxyapatite 0.25 and 0.75 g/cm^3^; Bruker) for measurements of BMD.

### Dynamic histomorphometry

Mice were injected with calcein (30 mg/kg, i.p.) on the first day of loading and alizarin (30 mg/kg, i.p.) on the last day of loading. The tibiae were embedded, sectioned, and analyzed by dynamic histomorphometry at Pharmatest (Turku, Finland). After dissection and fixation in formalin, the tibiae were dehydrated in increasing series of ethanol concentrations, defatted in xylene, and embedded in methyl methacrylate. Two hundred micrometer transverse sections from ∼37% of the total length of the tibia distally from the proximal articular surface were analyzed. All parameters were assessed using the OsteoMeasure histomorphometry system (OsteoMetrics, Atlanta, GA, USA) following the guidelines of the American Society for Bone and Mineral Research (44).

### Toluidine blue staining

The plastic-embedded blocks were cut at ∼37% of the total length of the tibia distally from the proximal articular surface and gently polished prior to gluing to a glass slide. An ∼100 µm thick section was cut and subsequently ground to a final thickness of 20 µm, using a diamond saw and grinding machines (Exakt, Norderstedt, Germany). The sections were stained with toluidine blue (45).

### Immunohistochemistry

Four micrometer sections from decalcified, paraffin-embedded bones from the 37% site of the total length of the tibia from the proximal end were used for detection of alkaline phosphatase (ALP)–positive cells. Sections were immunostained using a rabbit polyclonal anti-ALP antibody (ab97384; Abcam, Cambridge, United Kingdom) and a horseradish peroxidase-labeled anti-rabbit antibody using 3,3’-diaminobenzidine as substrate (K4003; Agilent, Santa Clara, CA, USA) according to the manufacturer’s description. Antigen retrieval was performed in citrate buffer (pH = 6.0) overnight at 60°C. Background staining was reduced by incubating the sections in 3% H_2_O_2_ diluted in methanol for 20 min. Sections were incubated for 2 h at room temperature with the antibody diluted 1:30 in protein-blocking solution (X0909; Agilent). Nuclei were counterstained with hematoxylin. All washing steps were performed in PBS.

### Quantitative RT-PCR

For RNA analyses of cortical bone, the diaphyseal part of tibia was flushed to remove bone marrow, and the cortical bone was stored in RNAlater (Qiagen) at −80°C until analysis. RNA was extracted by first homogenizing bone in Trizol reagent (Thermo Fisher Scientific, Waltham, MA, USA) using a tissue lyser (Qiagen). After centrifugation to remove cell debris, the Trizol homogenate was mixed with an equal volume of chloroform, centrifuged, and the aqueous phase recovered. The aqueous phase was mixed with an equal volume of 70% ethanol and added to an RNeasy spin column (Qiagen). Thereafter, the isolation followed the protocol from the RNeasy Mini Kit (Qiagen). Single-strand cDNA was synthesized using a High Capacity cDNA Reverse Transcription Kit (Thermo Fisher Scientific). Quantitative RT-PCR analyses were performed by using predesigned Taqman Assays and Taqman Fast Advance Master Mix (Thermo Fisher Scientific). The following predesigned RT-PCR assays were used for gene expression analysis: *Sp7* (Mm04209856_m1), *Alpl* (Mm00475834_m1), *Col1a1* (Mm00801666_g1), *Sost* (Mm00470479_m1) (Thermo Fisher Scientific). The housekeeping gene *18S* (4310893E; Thermo Fisher Scientific) was used as the endogenous control in all analyses.

### Raman spectroscopy

Raman spectroscopy was performed using a confocal Raman microscope (α300 R; WITec, Ulm, Germany) equipped with a 532 nm laser. Samples used were the same as for dynamic histomorphometry, but wet-polished using 800–400 grit silicon carbide paper. On the sample surface, the laser was focused down using a ×50 objective with a numerical aperture of 0.7. Spectra were collected in the 300–1800 cm^−1^ spectral range using an electron-multiplying charge-coupled device detector cooled to −60°C, behind a 600 mm^−1^ grating, in 15 µm × 15 µm regions of interest, ∼10–12 µm away from bone surface (BS) in the periosteal and endocortical regions and the geometrical center of the cortex (Supplemental Fig. S1) using an integration time of 2 s per pixel and pixel size of 5 µm × 5 µm. The Raman metrics investigated included mineral crystallinity, taken as the reciprocal of the full width at half maximum of the ν_1_ PO_4_^3−^ peak at ∼960/cm, the apatite-to-collagen ratio, also referred to as the mineral-to-matrix ratio (ν_2_ PO_4_^3−^/Amide III), and the carbonate-to-phosphate ratio (ν_1_ CO_3_^2−^/ν_2_ PO_4_^3−^). The integral areas were ν_2_ PO_4_^3−^ (∼410–460/cm), Amide III (∼1223–1303/cm), and ν_1_ CO_3_^2−^ (∼1052–1092/cm).

### Statistical analyses

All statistical results are presented as means ± sem. Linear regression model was used to determine peak load. An unpaired Student’s *t* test at each individual time point was used to monitor body weights throughout the experiment. The overall effect of diet (control *vs.* vitamin A), loading (nonloaded *vs.* loaded), and their interaction on bone was assessed using 2-way ANOVA with repeated measures to account for the left and right limbs being paired with each mouse. When significances were detected, Sidak’s multiple comparison test was used to evaluate the effect of diet (control *vs.* vitamin A) and loading (nonloaded *vs.* loaded). GraphPad Prism 7 statistical software v.7.02 (GraphPad Software, La Jolla, CA, USA) was used, with *P* < 0.05 considered statistically significant.

## RESULTS

### Vitamin A decreased the loading response in trabecular bone

**Figure 2:**
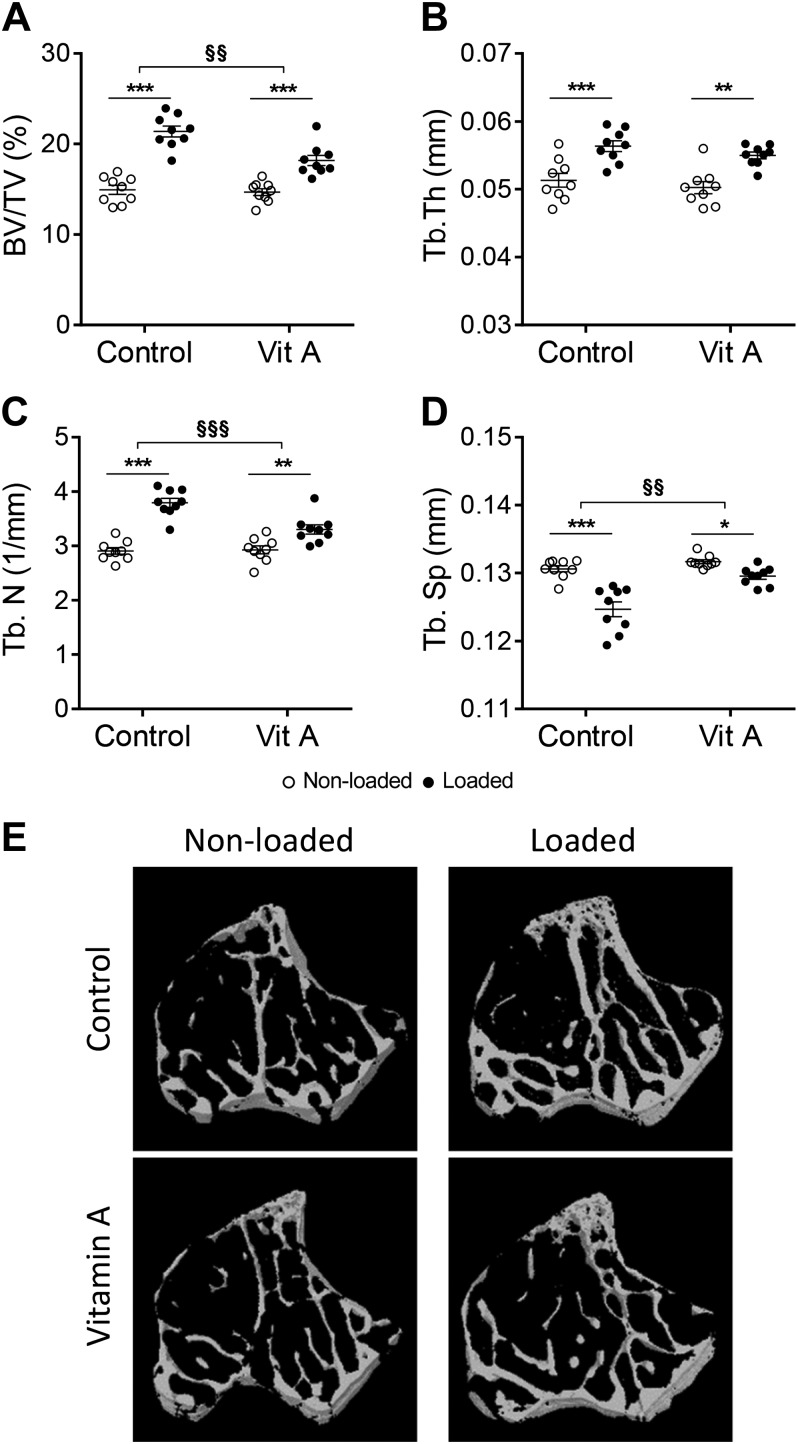
Vitamin A decreased the loading response in trabecular bone. BV/TV (*A*), trabecular thickness (Tb.Th) (*B*), trabecular number (Tb.N) (*C*), and trabecular separation (Tb.Sp) (*D*) as analyzed by µCT; representative images (*E*). Data in (*A*–*D*) are displayed as means ± sem, *n* = 9/group. *P < 0.05, **P < 0.01, ***P < 0.001 for loaded *vs.* nonloaded tibia of the same diet. *P* > 0.05 for vitamin A nonloaded *vs.* control nonloaded. ^§§^*P* < 0.01, ^§§§^*P* < 0.001 for the loading response in control *vs.* the loading response in vitamin A–treated mice.

Mice appeared healthy with no consistent effect of diet on body weight throughout the loading experiment (Fig. 1*C*). Two weeks loading in mice fed control diet resulted in an increase in bone volume per tissue volume (BV/TV) in the tibia by 43% (**Fig. 2*A, E***). Trabecular thickness was increased by 10% and trabecular number increased by 31%, leading to a significant decrease in trabecular separation of 5% (Fig. 2*B–D*). Vitamin A did not affect trabecular bone parameters in the tibia. The loading-induced effects on BV/TV, trabecular number and spacing (but not trabecular thickness) were significantly decreased in mice fed the vitamin A diet (Fig. 2*A, C–E*).

### Vitamin A decreased cortical bone and cortical bone loading response

**Figure 3:**
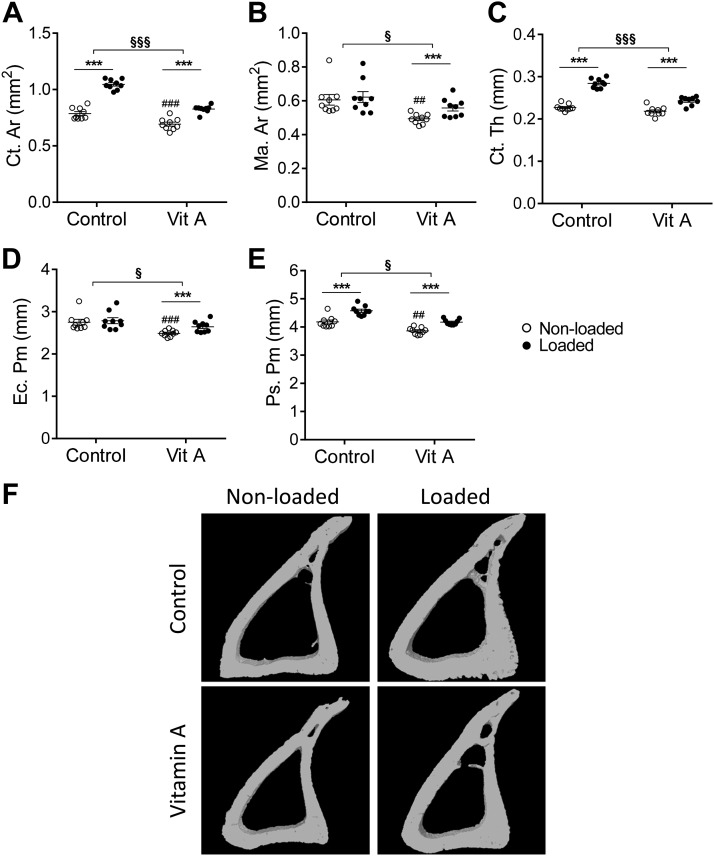
Vitamin A decreased cortical bone and cortical bone loading response. Cortical bone area (Ct.Ar) (*A*), marrow area (Ma.Ar) (*B*), cortical thickness (Ct.Th) (*C*), endocortical perimeters (Ec.Pm) (*D*), and periosteal perimeters (Ps.Pm) (*E*) as analyzed by µCT; representative images (*F*). Data are displayed as means ± sem, *n* = 9 per group. ***P < 0.001 for loaded *vs.* nonloaded tibia of the same diet. ^##^*P* < 0.01, ^###^*P* < 0.001 for vitamin A nonloaded *vs.* control nonloaded. ^§^*P* < 0.05, ^§§§^*P* < 0.001 for the loading response in control *vs.* the loading response in vitamin A–treated mice.

Loading alone increased cortical bone area and cortical thickness *via* increased periosteal perimeter, with no change in marrow area or endocortical perimeter (**Fig. 3**). Vitamin A diet alone decreased cortical bone area by 12%, marrow area by 19%, endocortical perimeter by 10%, and periosteal perimeter by 8% compared with nonloaded control (Fig. 3*A, B, D–F*). The loading response to cortical bone area, cortical thickness, and periosteal perimeter was significantly decreased with vitamin A diet (Fig. 3*A, C, E, F*).

### Vitamin A reduced the loading-induced increase in periosteal and endocortical bone formation

**Figure 4:**
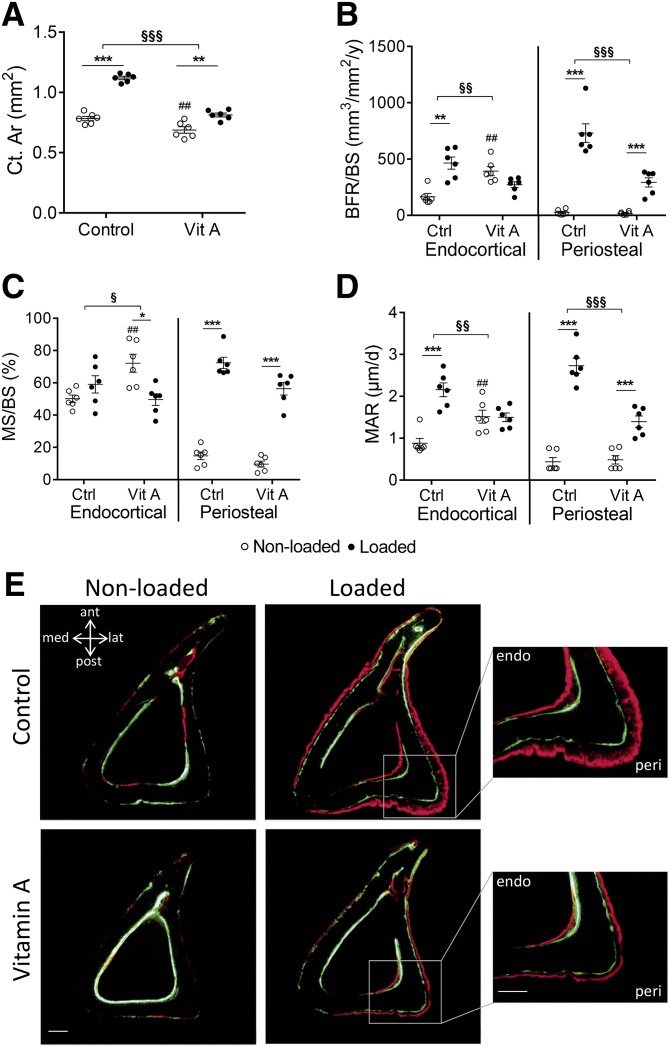
Vitamin A reduced the loading-induced increase in periosteal and endocortical bone formation. *A*) Cortical bone area (Ct.Ar). *B*) BFR/BS. *C*) MS/BS. *D*) MAR. Data are displayed as means ± sem, *n* = 6 per group. *P < 0.05, **P < 0.01, ***P < 0.001 for loaded *vs.* nonloaded tibia of the same diet. ^##^*P* < 0.01 for vitamin A nonloaded *vs.* control nonloaded. ^§^*P* < 0.05, ^§§^*P* < 0.01, ^§§§^*P* < 0.001 for the loading response in control *vs.* the loading response in vitamin A–treated mice. *E*) Representative sections with calcein (green) and alizarin (red); scale bars, 100 µm.

Histomorphometric analysis of cortical bone area (**Fig. 4*A***) reproduced the µCT results of cortical bone area (Fig. 3*A*), demonstrating an increase by loading and significant decrease of this response by vitamin A.

Loading increased periosteal and endocortical bone formation rate (BFR)/BS (+2640 and +182% respectively; Fig. 4*B, E*). Periosteally, this was due to an increase in both mineralized surface (MS)/BS (+391%, Fig. 4*C*) and mineral apposition rate (MAR; +524%, Fig. 4*D*) but only reflected by an increased MAR endocortically (+145%, Fig. 4*D*).

Vitamin A alone did not alter periosteal BFR/BS, MS/BS, or MAR (Fig. 4*B–E*). Endocortically, vitamin A alone increased BFR/BS by +138%, which was due to an increase in both MS/BS (+44%) and MAR (+72%) (Fig. 4*B–E*), resulting in decreased endocortical perimeter (−10%, Fig. 3*D*) and decreased marrow area (−19%, Fig. 3*B*).

Vitamin A treatment reduced the loading-induced increase in periosteal and endocortical BFR/BS, reflected by reduced increase in MAR at both sites (Fig. 4*B, D, E*). The loading-induced increase of MS at the periosteal surface was not significantly affected by vitamin A (Fig. 4*C*). Toluidine blue staining displays the newly formed bone (Supplemental Fig. S2).

### Vitamin A inhibited the increase in the expression of osteoblastic genes and ALP protein in response to loading

**Figure 5:**
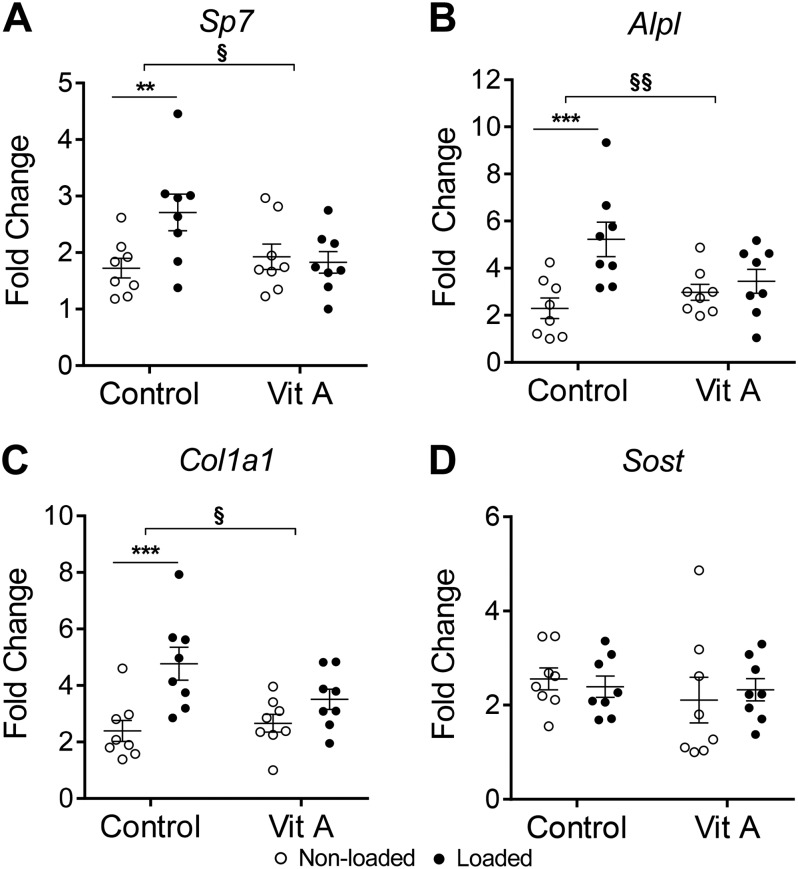
Vitamin A inhibits the increase in osteoblastic genes observed in response to loading. mRNA expression of *Sp7* (*A*), *Alpl* (*B*), *Col1a1* (*C*), and *Sost* (*D*) from the cortical bone of the tibia. Data are displayed as means ± sem, *n* = 8 per group. **P < 0.01, ***P < 0.001 for loaded *vs.* nonloaded tibia of the same diet. *P* > 0.05 for vitamin A nonloaded *vs.* control nonloaded. ^§^*P* < 0.05, ^§§^*P* < 0.01 for the loading response in control *vs.* the loading response in vitamin A–treated mice.

**Figure 6:**
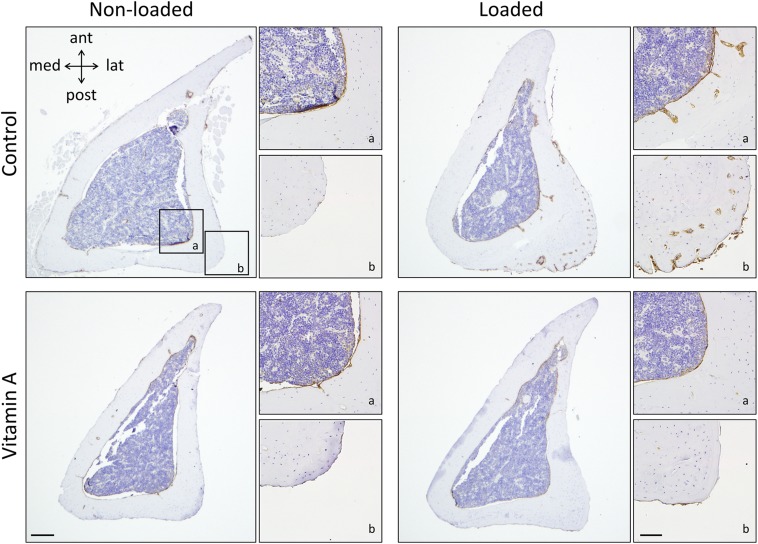
Vitamin A inhibits the loading-induced increase in ALP protein expression. Representative immunhistochemical staining for ALP in the tibia. Overview images of control and vitamin A loaded and nonloaded bones; scale bar, 100 µm. Magnified images in endocortical (*a*) and periosteal (*b*) regions; scale bar, 20 µm.

Loading increased the expression of osteoblastic genes *Sp7*, *Alpl*, and *Col1a1* (**Fig. 5*A–C***). These responses were significantly suppressed by vitamin A (Fig. 5*A–C*). No change in the expression of *Sost* was observed in response to loading or vitamin A diet (Fig. 5*D*). In line with the inhibited *Alpl* gene expression by vitamin A in the presence of loading, ALP protein expression was also reduced (**Fig. 6**). In nonloaded tibia from control and vitamin A mice, ALP-positive cells were present at the endocortical surfaces. In loaded bones from mice fed the control diet, cells robustly stained for ALP were present at both endocortical and periosteal surfaces, and also in islands of newly formed bone on both surfaces. This response was absent in loaded bones from mice fed the vitamin A diet.

### Raman spectroscopy indicates that in the presence of vitamin A, loading does not improve bone quality

**Figure 7:**
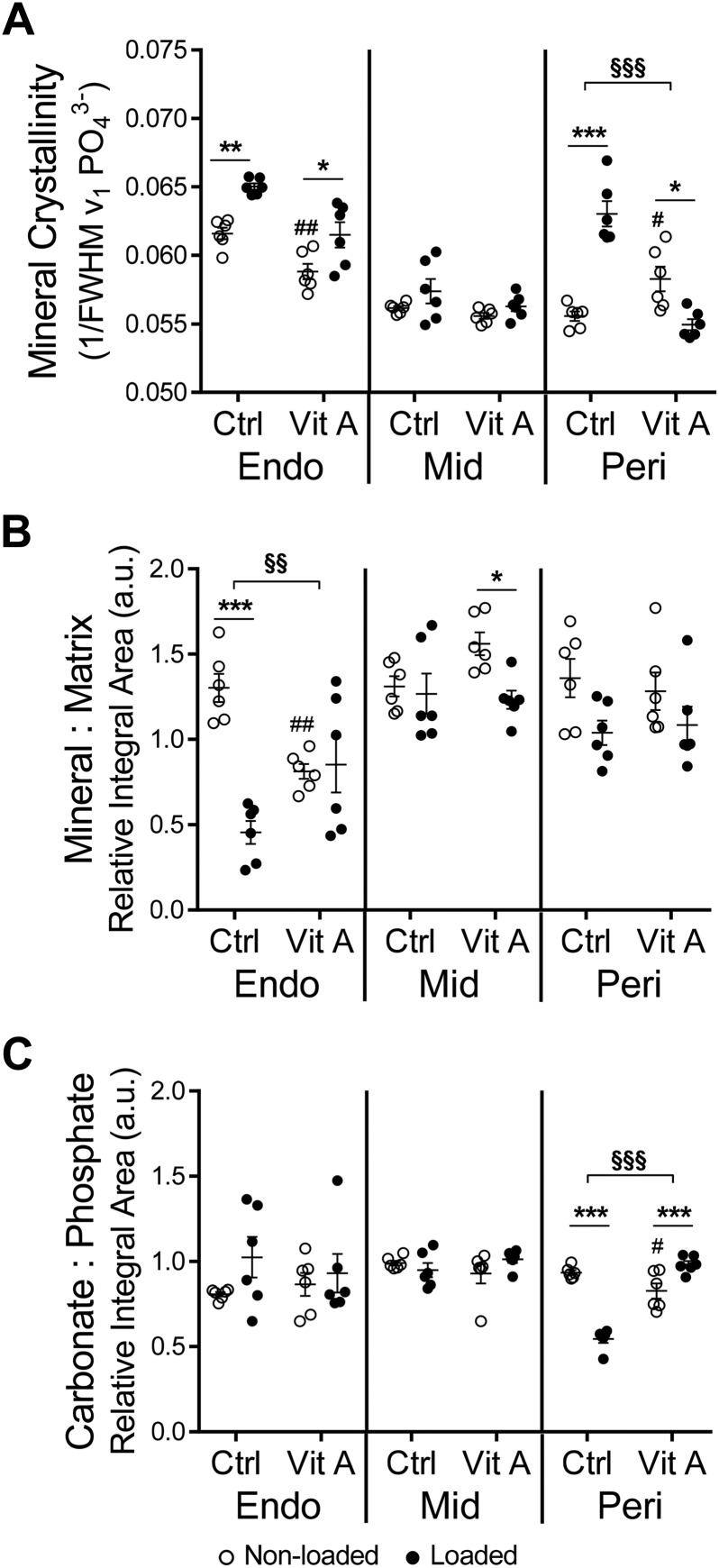
Raman spectroscopy indicates that in the presence of vitamin A, loading does not improve bone quality. Samples were analyzed at the endosteal (Endo) region, in the geometrical center of the cortex (Mid), and at the periosteal (Peri) region of the tibia (Supplemental Fig. S1) for mineral crystallinity (*A*), mineral-to-matrix ratio (*B*), and carbonate-to-phosphate ratio (*C*). Data are displayed as means ± sem, *n* = 6 per group. *P < 0.05, **P < 0.01, ***P < 0.001 for loaded *vs.* nonloaded tibia of the same diet. ^#^*P* < 0.05, ^##^*P* < 0.01 for vitamin A nonloaded *vs.* control nonloaded. ^§§^*P* < 0.01, ^§§§^*P* < 0.001 for the loading response in control *vs.* the loading response in vitamin A–treated mice.

Endocortically, loading increased mineral crystallinity regardless of diet, whereas the vitamin A diet decreased mineral crystallinity compared with nonloaded control (**Fig. 7*A***). At the same surface, the mineral-to-matrix ratio was reduced in response to loading in control mice (Fig. 7*B*). Vitamin A alone also reduced the endocortical mineral-to-matrix ratio compared with nonloaded control, and loading had no effect in mice fed the vitamin A diet (Fig. 7*B*). Endocortically, the ratio of carbonate to phosphate remained unchanged in response to loading, vitamin A diet, or both (Fig. 7*C*).

The composition of the central region of the bone remained unchanged by loading, vitamin A, or the combination of both (Fig. 7*A–C*).

Periosteally, loading increased mineral crystallinity in control mice (Fig. 7*A*). At the same surface, vitamin A alone increased mineral crystallinity compared with nonloaded control, and loading of vitamin A–treated bone resulted in decreased mineral crystallinity (Fig. 7*A*). The mineral-to-matrix ratio was unchanged periosteally with loading, vitamin A, or the combination (Fig. 7*B*). Loading decreased the carbonate-to-phosphate ratio periosteally (Fig. 7*C*). Vitamin A diet decreased carbonate-to-phosphate ratio, which increased when the bones were loaded (Fig. 7*C*).

## DISCUSSION

We show in the present study that the anabolic bone response to mechanical loading on trabecular and cortical bone mass is suppressed by a clinically relevant dose of vitamin A. Vitamin A decreased the amount of new bone formed in cortical bone because of inhibition of loading-induced bone formation on periosteal and endocortical surfaces. In addition, vitamin A, loading, and the combination thereof resulted in site-specific effects on the nanostructure of the newly formed bone. These data provide evidence that vitamin A negatively affects the enhancement of bone mass and quality in response to loading.

Mechanical loading increased trabecular bone mass, trabecular number, and thickness, but trabecular separation was decreased. Effects of hypervitaminosis A on trabecular bone have been varied, with some studies showing decreased trabecular bone (14, 46) and others claiming no effect observed (19, 21, 47, 48) in mice and rats. Our previous study, carried out using the same dose of vitamin A (RAE 60 µg/g chow) as used in the present study in mice treated for 4 and 10 wk, or using a hypervitaminosis A diet (RAE 450 µg/g chow) for 8 d, indicated no effect of vitamin A on trabecular bone in the long bones or the vertebrae (20), which is consistent with the µCT results in the present study. Importantly, however, although vitamin A did not affect trabecular bone alone, it was able to suppress the magnitude of the loading-induced decrease in trabecular spacing and increase in BV/TV. Although no dynamic histomorphometry in trabecular bone was performed, it is likely that this response is due to decreased osteoblast activity, similar to the observations in cortical bone.

Repeatedly, short-term treatment of rats with hypervitaminosis A (142- to 500-fold increased vitamin A intake) has been shown to decrease cortical bone mass (14, 15, 17). In the present study, 6 wk of a clinically relevant vitamin A diet (13-fold increased vitamin A content) given to mice also decreased cortical bone parameters, such as cortical area and endocortical and periosteal perimeters. The decreased endocortical perimeter was attributed to enhanced endocortical bone formation. This is in line with our previous studies, in which we observed that vitamin A decreased endocortical perimeter because of increased endocortical bone formation (20).

We show here that 6 wk of excess vitamin A diet decreases periosteal perimeter without an accompanying decrease in BFR. Previous hypervitamosis A studies have demonstrated inhibited periosteal osteoblast activity under basal conditions in long bones of rats (15) and pericranially in mouse calvaria (16). In agreement, we showed previously that vitamin A decreased periosteal perimeter in long bones of mice by an initial decrease of BFR after 8 d, which transiently disappears after 4 and 10 wk (20), indicating that we have passed the window of decreased periosteal BFR induced by vitamin A in the present study.

The novel finding in the present study is that vitamin A suppressed the loading-induced increase in cortical bone mass by suppressing bone formation. We observed, by dynamic histomorphometric assessment, that vitamin A suppressed the loading-induced increase in osteoblast activity (MAR), which led to decreased loading-induced periosteal and endocortical BFR. The increase in endocortical osteoblast number (MS) in response to vitamin A was lost when bones were loaded. Periosteally, vitamin A did not affect the loading-induced increase in the number of osteoblasts. Vitamin A exerts effects on cells by activation of retinoic acid receptors (RARs), using either RARα, RARβ, or RARγ in heterodimeric complexes with retinoid X receptors (23, 24, 49). All 3 RARs are present in bone tissue (50). These observations, together with our previous findings (20), indicate that functional retinoid receptors are expressed under physiologic conditions in both endocortical and periosteal osteoblasts. On the endocortical surface, these receptors appear to be expressed in both proliferating preosteoblasts and terminally differentiated osteoblasts. We cannot exclude, however, that the effects on osteoblasts may be due to extrinsic effects mediated by other cell types in the bone microenvironment expressing RARs, because it has recently been shown that deletion in mice of *Rarg* in *Prxx1*-expressing limb bud–derived mesenchymal stem cells results in not only decreased trabecular bone mass but also in effects on angiogenesis and B-lymphopoiesis (51). It seems that the decrease in cortical thickness observed in mice with global deletion of *Rarg* may also be caused by extrinsic effects, because this effect was not observed in mice with deletion of *Rarg* in *Prxx1*- or *Sp7*-expressing cells.

Importantly, under loading conditions, the retinoid receptors on endocortical surfaces inhibit proliferation of preosteoblasts and activity of mature osteoblasts, whereas the opposite is seen under physiologic conditions. On the periosteal surface it is not clear if the transient inhibitory effect on physiologic bone formation by vitamin A is due to increased number or activity of osteoblasts (20), but under loading conditions it is clear that inhibition of bone formation by vitamin A is due to decreased osteoblast activity rather than number. Taken together, these findings demonstrate that under physiologic conditions, vitamin A stimulates endocortical, but inhibits periosteal, osteoblast activity, and that under loading conditions, vitamin A inhibits both endocortical and periosteal osteoblast activity. In this context, it is interesting to note that heterotopic bone formation in different experimental models can be inhibited by 3 different RARγ agonists (52) and that the RARγ agonist palovarotene is now in clinical trials to prevent heterotopic bone formation in patients with the inherited disease fibrodysplasia ossificans progressiva (53).

The enhanced cortical bone formation induced by loading was associated with increased mRNA expression of *Sp7*, which is a master regulator of bone formation, *Alpl*, which encodes an osteoblastic enzyme involved in bone mineralization, and *Col1a1*, which encodes one of the proteins making up collagen type I fibers. Vitamin A alone did not alter the basal expression of these transcripts. This is in agreement with the observation that vitamin A did not affect periosteal osteoblast activity at the end of the experiment, but is in contrast with the increased osteoblast activity observed endocortically. A possible explanation may be that there are more periosteal than endocortical cells present in the RNA extracts. In line with this notion, vitamin A has been shown to increase the expression of osteoblastic genes (*Alpl*, *Runx2*) in extracts from periosteum-free cortical bone containing bone marrow, deducing that excess vitamin A increases endocortical and marrow osteoblast activity and mineralization (14). Vitamin A inhibited the loading-induced increase in osteoblastic genes and the expression of ALP protein on endocortical and periosteal surfaces. This is in line with the inhibition of osteoblast activity and bone formation on both cortical bone surfaces observed by dynamic histomorphometry. Interestingly, strong ALP staining is present in the holes mainly found at the periosteal site of bone formation, indicating site-specific active bone formation even after 2 wk of loading.

*Ex vivo*, vitamin A (all-*trans* retinoic acid) has been reported to downregulate several genes associated with osteoblast differentiation and function such as *Runx2*, *Sp7*, *Alpl*, *Bglap* (encoding osteocalcin), and *Col1a1* in calvarial bones (50). Several studies have been performed, aiming to investigate the effect by vitamin A *in vitro* on cell proliferation, differentiation, and bone-forming activity, using different cell lines and primary cells, different retinoids, and varying experimental design. The outcome of these studies has not demonstrated any consistent effects, with both inhibitory and stimulatory effects observed (23, 24, 49). Some of the discrepancies might be due to the dose used, with stimulatory effects generally seen at low concentrations and inhibitory at high, but there are also examples of stimulatory effects at high concentrations (24). It is also likely that the effect of retinoids depends on the stage of differentiation of the cells used (49). *In vivo* observations by us and others demonstrate that vitamin A has opposite effects on osteoblasts, depending on the localization and on environmental conditions (15, 16, 19, 20, 46) suggesting that the source of the cells and the design of the experiment will influence the effects obtained *in vitro*.

Mechanical loading improves bone structure on a macro and micro scale, leading to increased bone strength and decreased fracture risk (54–56). In contrast, hypervitaminosis A decreases bone structure on a macro scale, leading to decreased bone strength and increased fracture risk (8, 9, 14, 21, 22, 57). In response to loading, the mineral-to-matrix ratio was decreased endocortically but unchanged periosteally. This is in line with our previous study, showing that the periosteal mineral-to-matrix ratio was not affected by loading (54). Loading increased mineral crystallinity endocortically and periosteally, indicating larger crystal size and a better coalignment of the mineral particles. As a result, the new bone formed is more organized and may thus be more resistant to fractures. At the periosteal site, loading induced longer and thinner crystals, as illustrated by decreased carbonate-to-phosphate ratio and, as previously shown by small-angle X-ray scattering measurements, decreased mineral plate thickness by loading (54). Thus, the endosteum and periosteum respond differently to load at the nano scale. The difference in response to loading could be due to different strain levels at the 2 sites or to the different chemical and physical environments (endosteum-to-marrow interaction *vs.* periosteum-to-muscle interaction).

On the nano scale, vitamin A decreased endocortical mineral crystallinity and the mineral-to-matrix ratio without affecting the carbonate-to-phosphate ratio, confirming that new bone is formed endocortically. In contrast, the unaltered periosteal mineral-to-matrix ratio indicates that the nanomechanical competence of the bone is not affected by vitamin A. Just as loading alone, vitamin A alone decreased the carbonate-to-phosphate ratio and increased the mineral crystallinity periosteally. When combined, vitamin A blunted the loading-induced decrease in endocortical mineral-to-matrix ratio, confirming that the loading response is blunted by vitamin A endocortically. Interestingly, when combined with vitamin A, the loading-induced decrease in carbonate-to-phosphate ratio was blocked and the increase in mineral crystallinity was reversed, indicating that in the presence of vitamin A, loading does not improve bone quality on the periosteal site. It is important to note, however, that in the case of both nonloaded groups, very minimal newly formed bone exists at the periosteal and endocortical sites. Therefore, there is a risk that the measurements in fact reflect a combination of original bone and some newly formed bone.

It has been estimated that 30–50% of all women and 15–30% of all men will suffer from an osteoporotic fracture in their lifetime (58). Physical activity is frequently advised as a preventative approach against age-related bone loss. Apart from enhancements in BMD and bone strength, physical activity also promotes gain in muscle mass and muscle strength, thereby reducing the risk of falls and fractures altogether. These beneficial effects of exercise may be influenced by vitamin A.

The age of mice used in the present study are reflective of adults between 20 and 30 yr of age (32) and therefore represent individuals with peak bone mass. Mechanical loading of the tibia in mice may not necessarily be transferable to the strain applied in humans during physical activity. The action of the muscle-to-bone interaction has also been unexamined in the present study and may contribute to the loading effect observed on bone parameters. Dynamic histomorphometry allowed for quantification and visualization of the bone-forming response to loading. This is under the assumption that the section analyzed is representative of the entire specimen. Data from analysis provided a mechanism for reduced bone formation observed with excess vitamin A intake.

In summary, using a model of rapid bone formation, we show that a vitamin A dose of RAE 60 µg/g chow for 6 wk can suppress the anabolic bone response to mechanical loading in female C57BL/6 mice because of an effect that can be attributed mainly to decreased osteoblast activity.

## Supplementary Material

This article includes supplemental data. Please visit *http://www.fasebj.org* to obtain this information.

## Abbreviations

ALP: alkaline phosphatase
BFR: bone formation rate
BMD: bone mineral density
BS: bone surface
BV: bone volume
MAR: mineral apposition rate, MS, mineralized surface
µCT: microcomputed tomography
RAE: retinol activity equivalents
RAR: retinoic acid receptor
TV: tissue volume
