# Supplementary material for: Vitamin A decreases the anabolic bone response to mechanical loading by suppressing bone formation
Source: FASEB J. 2019 Jan 22;33(4):5237–47. doi: 10.1096/fj.201802040R (PMC6436664; doi:10.1096/fj.201802040R)
Supplement: Supplementary file 1 [file fj.201802040R.sf1.docx]

**D**

**Supplemental Figure 1: Regions of interest for Raman spectroscopy.** Images acquired using backscattered electron scanning electron microscopy (BSE-SEM) at low vacuum, 20kV accelerating voltage and ~66.7 Pa water vapour pressure (Quanta 200 environmental SEM. FEI Company, The Netherlands). C: Peri = periosteal, Mid = geometrical centre of the cortex, and Endo = endocortical. Scale bars in A = 500µm, B = 100µm, and C = 50µm. D: Typical Raman spectrum of bone. Integral areas for 𝜈_2_ PO_4_^3-^ (410–460 cm^-1^), 𝜈_1_ CO_3_^2-^ (1052–1092 cm^-1^), and amide III (1223–1303 cm^-1^) are highlighted. Inset: Mineral crystallinity is taken as the inverse full-width at half-maximum (FWHM^-1^) 𝜈_1_ PO_4_^3-^.
