# Supplementary material for: Vitamin A decreases the anabolic bone response to mechanical loading by suppressing bone formation
Source: FASEB J. 2019 Jan 22;33(4):5237–47. doi: 10.1096/fj.201802040R (PMC6436664; doi:10.1096/fj.201802040R)
Supplement: Supplementary file 2 [file fj.201802040R.sf2.docx]

**Supplemental Figure 2: Toluidine blue staining illustrates newly formed bone in response to loading.** Representative images of toluidine blue staining in the tibia. Overview images of control and vitamin A loaded and non-loaded bones. Scale bars represent 100µm.

endo

peri

ant

lat

post

med

Non-loaded

Loaded

Control

Vitamin A

endo

peri
